# Resorcinol–Formaldehyde-Derived Carbon Xerogels: Preparation, Functionalization, and Application Aspects

**DOI:** 10.3390/ma16196566

**Published:** 2023-10-05

**Authors:** Grigory B. Veselov, Aleksey A. Vedyagin

**Affiliations:** Boreskov Institute of Catalysis, 5 Lavrentyev Ave., 630090 Novosibirsk, Russia

**Keywords:** carbon xerogel, sol–gel synthesis, functionalization, metal doping, application

## Abstract

Carbon xerogels (CXs) are materials obtained via the pyrolysis of resins prepared via the sol–gel polycondensation of resorcinol and formaldehyde. These materials attract great attention as adsorbents, catalyst supports, and energy storage materials. One of the most interesting features of CXs is the possibility of fine-tuning their structures and textures by changing the synthesis conditions in the sol–gel stage. Thus, the first part of this review is devoted to the processes taking place in the polycondensation stage of organic precursors. The formation of hydroxymethyl derivatives of resorcinol and their polycondensation take place at this stage. Both of these processes are catalyzed by acids or bases. It is revealed that the sol–gel synthesis conditions, such as pH, the formaldehyde/resorcinol ratio, concentration, and the type of basic modifier, all affect the texture of the materials being prepared. The variation in these parameters allows one to obtain CXs with pore sizes ranging from 2–3 nm to 100–200 nm. The possibility of using other precursors for the preparation of organic aerogels is examined as well. For instance, if phenol is used instead of resorcinol, the capabilities of the sol–gel method become rather limited. At the same time, other phenolic compounds can be applied with great efficiency. The methods of gel drying and the pyrolysis conditions are also reviewed. Another important aspect analyzed within this review is the surface modification of CXs by introducing various functional groups and heteroatoms. It is shown that compounds containing nitrogen, sulfur, boron, or phosphorus can be introduced at the polycondensation stage to incorporate these elements into the gel structure. Thus, the highest surface amount of nitrogen (6–11 at%) was achieved in the case of the polycondensation of formaldehyde with melamine and hydroxyaniline. Finally, the methods of preparing metal-doped CXs are overviewed. Special attention is paid to the introduction of a metal precursor in the gelation step. The elements of the iron subgroup (Fe, Ni, Co) were found to catalyze carbon graphitization. Therefore, their introduction can be useful for enhancing the electrochemical properties of CXs. However, since the metal surface is often covered by carbon, such materials are poorly applicable to conventional catalytic processes. In summary, the applications of CXs and metal-doped CXs are briefly mentioned. Among the promising application areas, Li-ion batteries, supercapacitors, fuel cells, and adsorbents are of special interest.

## 1. Introduction

Nowadays, carbon nanomaterials are of significant interest due to their unique properties. The list of areas in which carbon materials are applied is constantly expanding.

In recent years, there have been an increasing number of studies on developing new strategies for the synthesis of carbon materials. For instance, the electrolysis of CO_2_ with the formation of carbon is regarded as both an environmentally friendly route to produce carbon materials and a new CO_2_ capture technology [1,2]. However, this approach is poorly capable of fine-tuning the structure and properties of carbon materials. A significant number of contributions are devoted to the template synthesis of ordered microporous, mesoporous, and macroporous carbons [3]. Great possibilities for the controllable synthesis of new carbonaceous material are opened if metal–organic frameworks, such as ZIF-8, are used as precursors [4]. On the other hand, sol–gel-derived carbon materials, such as carbon xerogels (CXs), aerogels, and cryogels, attract special attention. Usually, they are synthesized by the pyrolysis of organic gels obtained by the sol–gel polycondensation of organic monomers such as resorcinol and formaldehyde [5]. Carbon xerogels and aerogels have high porosity and a developed surface area, and their textural properties can be fine-tuned by selecting the appropriate sol–gel synthesis conditions [6]. In addition, CXs can be easily molded (for example, in the form of monoliths, thin films, and granules), which is important for their practical use [7,8]. The structure of these materials is represented by a network of nanoscale primary particles. Therefore, they can possess micro-, meso-, and macroporosity: micropores are formed inside primary particles, while larger pores are formed in the voids between them. Thus, effective salt-resistant solar-driven interfacial evaporation systems based on carbon aerogels were reported recently [9]. Despite their high porosity, CXs have a sufficiently high electrical conductivity, which enables their use in energy storage devices [10]. Carbon aerogels demonstrate high electromagnetic interference shielding and thermal insulation performance as well [11].

It should be noted that RF gels and other types of organic gels could be used to produce ordered mesoporous carbon nanomaterials via silica-templating routes [3] or hollow carbon nanostructures of the desired size and shell thickness via the pyrolysis of metal–organic frameworks [4]. Contrarily, the structure of pristine RF-derived CXs and aerogels is less spatially ordered. At the same time, the control of pH and other parameters still allows one to produce these materials with predefined textural characteristics.

By adjusting the chemical composition of their surfaces and textural properties, suitable supports can be obtained for use in catalytic and electrocatalytic processes, as well as effective adsorbents for the elimination of oils and transition-metal ions [12]. In particular, it has been shown that the pore texture of carbon gels can be adjusted so that diffusion constraints are minimized [13]. In addition, the surface of CXs can be modified by various methods, including the use of different precursors during the synthesis of the organic gel [14,15] or subsequent treatment in either the gas or liquid phase [16,17]. In addition to conventional impregnation-based methods, the active component can be introduced into the composition at the sol–gel polymerization step.

**Figure 1:**
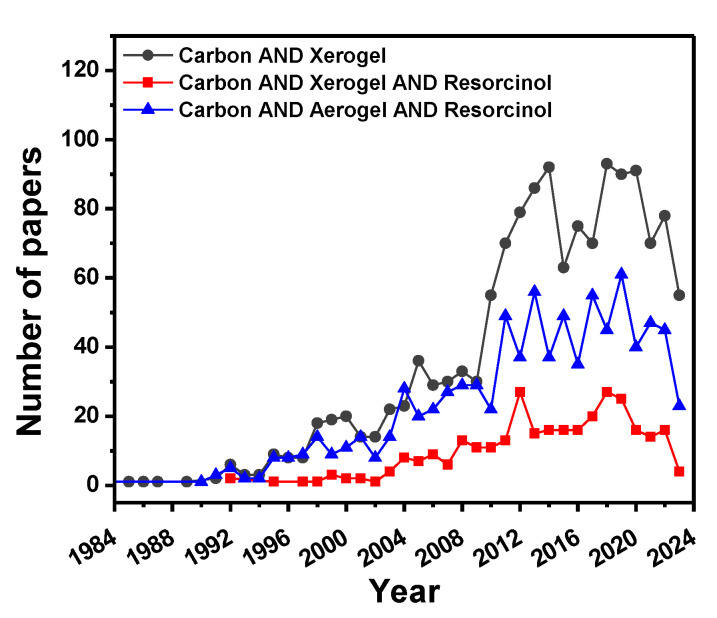
Number of publications in the SciFinder^n^ database related to carbon xerogels and aerogels.

Due to their unique properties, CXs attract great attention from researchers. According to the SciFinder^n^ database (Figure 1), the greatest interest in these materials began in 2010-2012 and has not faded since then. As is seen, materials synthesized from resorcinol/formaldehyde polymer precursors account for a significant portion of publications on carbon xerogels and aerogels.

The purpose of this paper is to review recent advances in the preparation of CXs in order to reveal some correlations between the preparation conditions and the properties of CXs. Firstly, the processes occurring at the polycondensation stage of organic precursors are considered. Then, aspects of the influence of sol–gel synthesis conditions on the texture of obtained materials are summarized and analyzed. The methods of gel drying, as well as pyrolysis conditions, are reviewed as well. Further, methods of modifying the surfaces of CXs by introducing oxygen-containing functional groups and other heteroatoms are analyzed. Special attention is paid to the preparation of metal catalysts based on CXs. At the end, a brief overview of the applications of CXs is provided.

## 2. Sol–Gel Synthesis of Nanostructured Materials: From Oxides to Carbon Xerogels and Aerogels

### 2.1. General Features of the Sol–Gel Process

The sol–gel method is known to be one of the most promising methods for the synthesis of oxide systems. Many compounds conventionally used as adsorbents and catalyst supports, such as ZrO_2_ [18], TiO_2_ [19], MgO [20], Al_2_O_3_ [21], MgAl_2_O_4_, and SiO_2_ [22], can be obtained using this technique. The textural characteristics of these materials have a decisive influence on the effectiveness of their application. Thus, the catalyst support must have an optimal specific surface area (SSA) and porous structure to effectively enable the transport of the reactant to the particles of the active component. The sol–gel method enables produced compounds to be obtained in a highly dispersed state and provides a wide range of possibilities for regulating their textural properties.

**Figure 2:**
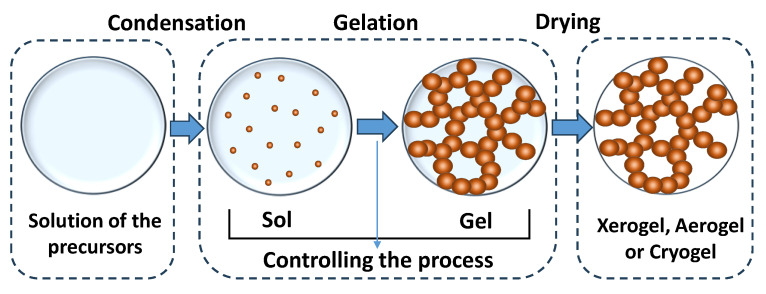
A principal scheme of the sol–gel synthesis of nanomaterials.

A principal scheme for the synthesis of materials by the sol–gel method is shown in Figure 2. In the first stage of synthesis, the precursor molecules condense to form dispersed sol particles. The coarsening of sol particles leads to their agglomeration and the formation of gels. In this case, individual particles are bound in branched chains, and the solvent fills the space between them. The solvent is then removed and, if necessary, further heat treatment (calcination) is carried out.

It is worth noting that the properties of gels are determined by the conditions under which polycondensation is carried out, namely, the pH value, temperature, the concentrations of precursors, and their ratio [23,24,25]. These parameters affect the ratio between hydrolysis and polycondensation rates, which significantly influences the particle size. Since the size of the primary particles, as a rule, is preserved during the drying process, the variation in the sol–gel synthesis conditions allow one to control the properties of the resulting materials.

There are several ways to remove the solvent from the gel structure [26]:

- Thermal drying under atmospheric pressure conditions to obtain *xerogels*. In this case, there is a significant loss of porosity during drying due to the influence of capillary forces.
- Cyclic freezing/heating to produce materials called *cryogels*.
- Drying under supercritical conditions, which are the gentlest for the porous structure. Supercritical fluids have zero surface tension, which contributes to the preservation of porosity. The *aerogels* formed in this way have the most developed surface and the largest pore volume. At the same time, the structure of aerogels may be more brittle and have less stability at high temperatures.

### 2.2. Sol–Gel Synthesis of Organic and Carbon Xerogels

Being widely used for the synthesis of various oxide materials, in recent decades, the sol–gel method has been extended to the production of carbon materials. The synthesis of carbon aerogels was first reported by Pekala in 1989 [5]. Resorcinol and formaldehyde were used as precursors in this work. The main stages of the process correspond to those of the production of silica gel and other similar materials. It has been found that at slightly acidic pH values, resorcinol and formaldehyde undergo polycondensation in aqueous solutions to form gels, which can be dried under supercritical conditions to produce aerogels having a specific surface area of 500–700 m^2^/g.

**Figure 3:**
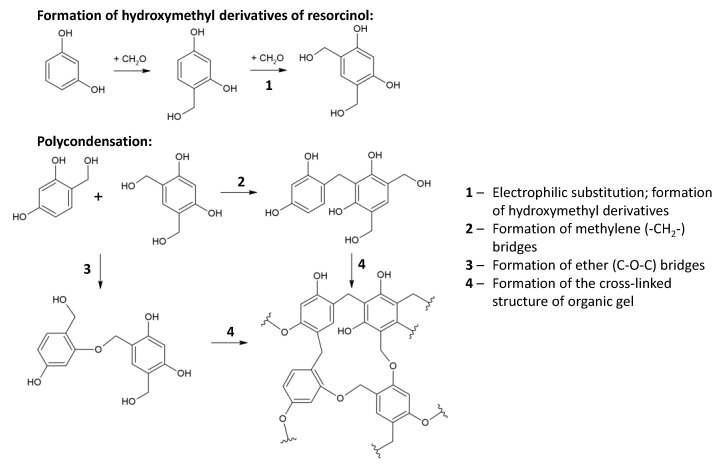
General processes taking place during the polycondensation of resorcinol with formaldehyde.

The polycondensation reaction takes place according to the scheme shown in Figure 3. During the process, the following stages occur: (1) the formation of hydroxymethyl derivatives of resorcinol by an electrophilic substitution reaction; (2) the condensation of hydroxymethyl derivatives to form -CH_2_- and -CH_2_-O-CH_2_- bridges; and (3) the disproportionation of ether bridges (-CH_2_-O-CH_2_-) to form methylene bridges (-CH_2_-) and the release of formaldehyde molecules. The high reactivity of resorcinol in the electrophilic substitution reaction is facilitated by the increased electron density at positions 2, 4, and 6 of the aromatic ring. Although the highest electron density is achieved at position 2, the presence of steric hindrance leads to the reaction mainly taking place at positions 4 and 6. Although this process is possible over a wide range of pH values, values ranging from 6.5 to 7.4 are reported [5]. Since the aqueous solution of resorcinol produces an acidic reaction, sodium carbonate is added to achieve the required pH values.

The kinetic features of the process have been studied as well [5]. The reaction rate has been shown to depend on the concentration of the sodium carbonate catalyst. The pH of the medium in which the reaction is carried out is of key importance: i.e., sodium carbonate acts as an agent for controlling this value. It was found that within the first three hours, the reaction is of the first order toward formaldehyde, and during this time, 60% of formaldehyde manages to react. It was suggested that small resorcinol–formaldehyde (RF) clusters of 7–10 nm are formed during the first three hours. Then, the rate of the gel-forming process is mainly determined by the slower binding processes of these clusters to each other.

This approach to synthesizing carbon organic aerogels and xerogels has been used and further developed in the works of other researchers. They studied the influence of the main parameters of the sol–gel process on the properties of the materials obtained, investigated the pyrolysis conditions necessary for the production of microporous CXs, and developed methods for introducing heteroatoms and metals into their structures. All of these aspects will be discussed in the next sections.

## 3. Effect of the Synthesis Conditions on the Textural Properties of Carbon Xerogels and Aerogels

### 3.1. Polycondensation/Gelation Stage

**Figure 4:**
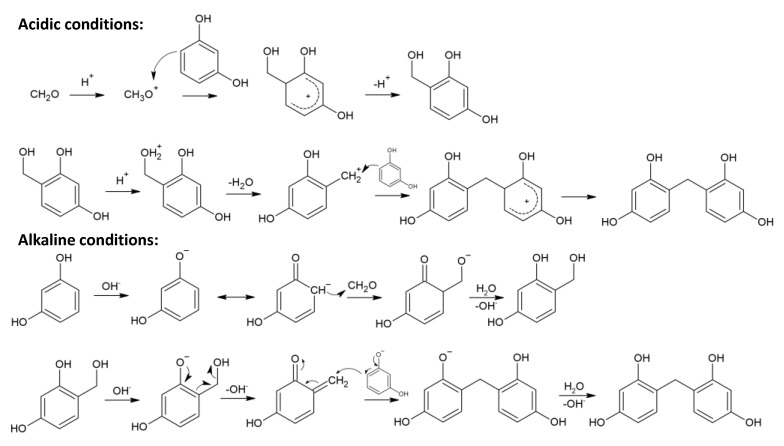
Mechanisms of the formation of hydroxymethyl derivatives of resorcinol and their polycondensation under acidic and alkaline conditions.

In this section, the effects of sol–gel synthesis conditions are considered in detail. It is obvious that the acidity of the medium has a significant effect on the rates of processes occurring during the polycondensation of resorcinol and formaldehyde. Thus, the reactivity of resorcinol ions formed due to the cleavage of the proton from the OH group in reaction with formaldehyde is significantly higher than that of the neutral molecules. An increase in pH results in a higher concentration of phenolate ions. Meanwhile, the electrophilic substitution reaction can also be catalyzed by H^+^ ions, since the protonation of the formaldehyde molecule increases its electrophilicity (Figure 4). The condensation processes of hydroxymethyl derivatives can also be catalyzed in acidic and alkaline media [27,28,29]. It is believed that in an alkaline medium, polycondensation occurs with the formation of quinone methide intermediates, which was recently confirmed by calculation methods for the case of the condensation of phenol with formaldehyde [30,31]. The gel formation time is the shortest either under strongly acidic conditions (pH = 1) or under conditions close to neutral (pH = 6–7) [27]. It is worth noting that the pH range of 5.5–7 is most often used for the synthesis of organic xerogels since such conditions enable the production of meso- and macroporous materials.

**Figure 5:**
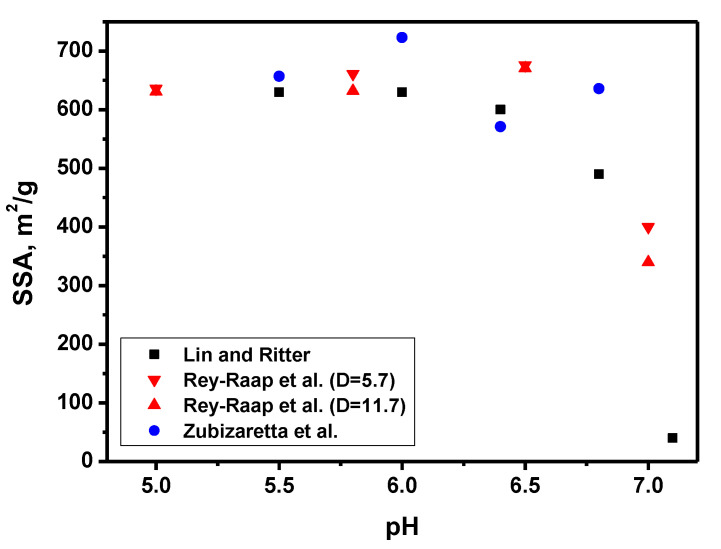
Effects of pH on the SSA values of CXs reported by Lin and Ritter [32], Rey-Raap et al. [34], and Zubizaretta et al. [35].

The effect of pH at the stage of the polycondensation of resorcinol and formaldehyde on the properties of the obtained CXs was studied by Lin and Ritter [32]. They used a resorcinol/formaldehyde ratio of 1/2, which corresponds to stoichiometry. It is worth noting that, since the polycondensation process is carried out at elevated temperatures (70–90 °C), hereinafter, the pH value of the precursor solution measured at room temperature is referred to. The acidity of the medium changes slightly during the process, so it is convenient to consider the initial pH value. Materials obtained at pH greater than 7 are practically non-porous, and their pore volume and specific surface area are close to zero (Figure 5). Apparently, at high pH, the structure of organic xerogels is less rigid, since the crosslinking processes between RF clusters responsible for the formation of the structure occur to a lesser extent. This results in the shrinkage of the porous structure in the pyrolysis stage. With a decrease in pH, a sharp increase in textural characteristics occurs, while the specific surface area reaches a maximum in the range of 5.4–6, but the pore volume changes monotonously throughout the studied pH range. This indicates that in the pH range of 5.4–6, larger pores (macropores) are formed that do not significantly contribute to the specific surface area. A further decrease in pH results in samples containing macropores only. These effects result primarily from the different sizes of the primary sol particles in the synthesis step. Thus, with an increase in the concentration of K_2_CO_3_ during the synthesis of RF gels, the size of the primary particles falls [33], which leads to a smaller diameter of pores formed as a result of the arrangement of these primary particles into a three-dimensional matrix of xerogels.

The most comprehensive study of the textural characteristics of organic xerogels obtained at various pH values and precursor concentrations was carried out by Rey-Raap et al. [36]. As a measure of the concentration of precursors, the value of the “dilution ratio” (D) was used. D is the ratio of the solvent substance amount to the total amount of resorcinol and formaldehyde substances. It was found that gel formation is possible only in a strictly defined pH range. It has been shown that the volume of micropores increases with increasing pH and D, as these conditions contribute to the polycondensation reaction. The maximum volumes of mesopores and macropores are observed in opposite pairs of pH-D parameters, which indicate that higher values of the volume of micropores are accompanied by lower values of the volume of macropores and vice versa. In the studied pH-D range, organic xerogels with an average pore size of 2 nm to 1000 nm can be obtained. After pyrolysis, this range narrows to 10–80 nm [37]. A visual validation of the obtained dependencies was performed using scanning electron microscopy [34]. In general, higher pH values yield smaller particles and higher D values result in higher volumes between these particles. However, the higher D values lead to a more fragile structure, so shrinkage may occur in certain cases, depending on the pH value. It is noted that additional possibilities for a controllable change in texture are also opened by varying the resorcinol/formaldehyde ratio.

The kinetics of the early stages of the formation of primary particles in RF gels in the presence of sodium carbonate were studied via Dynamic Light Scattering measurements [38]. It was shown that the size of freely diffusing primary clusters is independent of both reactant and carbonate concentrations at a given temperature, reaching the mean hydrodynamic radius of several nanometers before further changes were observed. However, at higher carbonate concentrations or reactant concentrations, more primary clusters are produced due to faster reactions. These primary particles eventually form crosslinked structures due to coalescence, agglomeration, and further polycondensation.

Another factor affecting the sol–gel polycondensation process is the presence of methanol. Indeed, methanol is present in all commercially available solutions of formaldehyde and acts as a stabilizing agent. The effect of the methanol concentration was studied by Alonso-Buenaposada et al. [39]. As is reported, a lower concentration of methanol produces samples with higher porosity. The exact effect is defined by other conditions, such as pH. In certain cases, the texture can be tuned from mesoporous to macroporous just by changing the methanol content from 12% to 0%. Therefore, researchers should be aware that the methanol content in commercial formaldehyde solutions significantly affects the final properties of CXs. Other alcohols can also be used for texture tuning. For instance, variation in the ethanol content during the synthesis of RF gels results in pore size values ranging from 1–2 nm to 60 nm [40].

Although the effect of pH on the texture of CXs has already been discussed, it is known that not only the pH but also the type of basic catalyst used plays an important role. Job et al. [41] compared the porosity of CXs synthesized using various alkaline (MOH, M = Li, Na, K) and alkaline-earth (M(OH)_2_, M = Ca, Ba, Sr) metal hydroxides. It was determined that the alkaline-earth bases significantly increase the pore size and pore volume at the same pH, which is due to the more effective decrease in electrostatic screening by cations with a charge of +2. This leads to a faster polymerization-induced phase separation and, as a consequence, a lower degree of crosslinking. Contrarily, the size of a metal cation was not shown to affect the sol–gel process. Horikawa et al. [33] claimed that the type of anion is also important. Therefore, for the same R/C ratio, hydrocarbonates (NaHCO_3_ and KHCO_3_) yield higher pore sizes than carbonates (Na_2_CO_3_ and K_2_CO_3_). However, the pH values of the precursor solutions were not considered in this research. Indeed, at the same R/C ratio, hydrocarbonates should produce lower pH values compared to carbonates, which, as mentioned before, yields products with larger pores. In another work by Calvo et al. [42], five basic compounds were considered: NaOH, Ca(OH)_2_, Na_2_CO_3_, NaHCO_3_, and Li_2_CO_3_. It was found that at the same pH, NaOH and Ca(OH)_2_ generate materials with similar porosity in comparison with Na_2_CO_3_, NaHCO_3_, and Li_2_CO_3_. The carbonates and hydrocarbonates yield materials with wider pore size distributions and larger pores. However, this result contradicts the previously mentioned work by Job et al. [41]. Overall, the mentioned papers indicate that during the synthesis of CXs, it is important to consider not just the pH value but the type of basic agent used as well.

Another approach that is interesting for consideration is the use of organic bases to avoid the presence of alkali metals. Recently, one such base, triethylamine, was applied to synthesized CXs [43]. The obtained materials were strictly microporous and consisted of large spheres 3–5 µm in size. In this case, the R/C ratio was quite high (1000–3000), which led to relatively low pH values. Again, the exact pH values are not mentioned in this paper. Nevertheless, the hydrogen storage capacity of such materials was as high as 4 wt%.

Temperature is known to affect the rate of all chemical reactions, and the process of the polycondensation of resorcinol and formaldehyde is not an exception. The effects of temperature in the gel-forming stage and the aging time of gels on the texture of the obtained organic xerogels were studied by Job et al. [44]. As is reported, the aging time has practically no effect on the volume and size of the pores if the polycondensation reaction reaches equilibrium. The time required to achieve equilibrium mainly depends on the synthesis temperature: at 50 °C, stabilization does not occur even after 72 h, regardless of pH, while at 70 and 90 °C, 24 and 48 h are required, respectively. The size and volume of the pores depend on the polycondensation temperature, especially at lower pH. The volume of the pores usually decreases with an increase in temperature, but this effect can be balanced by an even greater decrease in pH, which, as mentioned earlier, leads to an increase in the volume of the pores. At high temperatures, bubbles in the gels can also be formed, which leads to the formation of voids in the resulting monoliths. Therefore, a gel-forming temperature of 70 °C is preferable for a large-scale synthesis, even though higher temperatures contribute to the acceleration of polycondensation processes.

### 3.2. Inverse Emulsification Process

**Figure 6:**
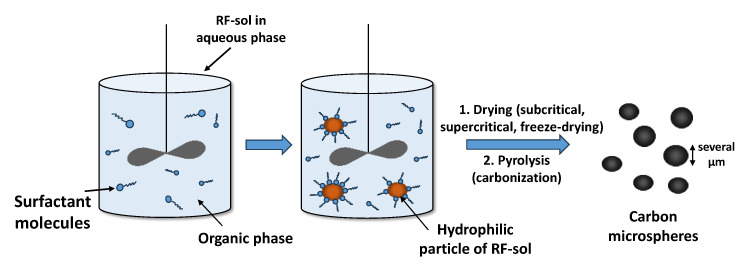
Scheme for production of carbon microspheres by emulsion procedure.

Another variation in the synthesis of materials based on RF gels allows for obtaining carbon microspheres. The inverse emulsification process is widely used to prepare particles by the sol–gel method and to control the surface morphology of CXs to comply with various applications [45]. The emulsion consists of two phases, which are insoluble in each other. One phase, called a dispersed phase, is represented by particles that are distributed in another continuous phase, called a dispersion medium. In emulsion polymerization, the hydrophobic monomer polymerizes with surfactants and a water-soluble polymerization initiator. In contrast, the inverse emulsification (IE) process involves the polymerization of a hydrophilic monomer, which occurs in a continuous organic phase using a surfactant, as shown in Figure 6.

According to the IE technique, polycondensation is carried out with the participation of water-soluble monomers, a non-polar organic solvent (dispersion medium), and an emulsion-stabilizing surfactant. Before the preparation of the emulsion, the preliminary polycondensation of resorcinol and formaldehyde is performed in the water phase, and then the obtained sol is converted into the organic phase. The interfacial surface tension at the boundary of the dispersed phase and the dispersion medium is minimized by surfactant molecules, which leads to the formation of spherical particles. Drying, followed by pyrolysis, leads to three different types of particles of carbon material, depending on the type of drying procedure.

However, by varying different parameters of the IE process (pH, concentration, amount of catalyst, mixing time, and type of surfactant), it is possible to obtain various types of morphologies, including porous, hollow, or non-porous microspheres, microcapsules, and fractal-like structures. Thus, it was shown [46] that the diameter of microspheres can be varied in a range of 5–50 microns by changing the pH and stirring time. An increase in the concentration of the Span-80 surfactant from 1 to 4 vol% resulted in smaller spherical particles with a narrower size distribution. Even higher concentrations of surfactant (10–50 vol%) lead to the formation of fractal-like structures resembling “bushes” and “flowers”. In some cases, microspheres have a developed porous structure containing micro- and mesopores. Such materials can be obtained by using supercritical drying and sequential freezing/heating drying methods [47,48,49].

The fields of application of carbon microspheres include supercapacitors, electrocatalysis, and such traditional catalytic processes as the reduction of nitrogen oxides by ammonia [48,50,51,52,53].

### 3.3. Drying Stage

The next stage of synthesis is the drying stage. As already noted, the removal of the solvent while maintaining the porous structure is a challenge. In order to mitigate the possible effects of drying pore shrinkage, several approaches have been developed:

- Step drying, e.g., 60 °C (24 h) → 80 °C (24 h) → 100 °C (24 h) → 120 °C (24 h) [54].
- Drying under reduced pressure (~1000 Pa), which allows one to reduce the drying time to ~20 h [35].
- Convection drying at a constant temperature of 70 °C. In this case, the removal of the solvent is accelerated due to the presence of airflow passing through the sample at a flow rate of 1–2 m/s [44].
- Microwave drying, which allows for removing the solvent in ~30 min [55,56]. Microwave radiation can be applied not only during drying but also during the gelation process. In this case, it affects porosity but still allows the precise tuning of textural properties [57].
- The exchange of the solvent for acetone before thermal drying. The lower surface tension of acetone reduces the loss in porosity [58].
- The exchange of the solvent for acetone and then for carbon dioxide and the removal of carbon dioxide under supercritical conditions. This is the most time-consuming and expensive method, but it allows for obtaining aerogels with the largest pore volume and specific surface area [5].

The time required to remove the solvent by convection drying depends mainly on the pore size of the sample [44]. Thus, at a drying temperature of 70 °C and an airflow rate of 2 m/s, the time required to remove 90 wt% of the solvent is 1 h at a pore size of 400–600 nm, 2.5 h at a pore size of 50 nm, and 3 h at a pore size of 15–20 nm. The removal of the remaining 10% of the solvent probably takes at least 8 h for samples with small pores. As reported, this stage can be accelerated by increasing the temperature after removing 90% of the solvent.

It is worth noting that in order to use CXs on an industrial scale, the development of large-scale synthesis methods is required. Thus, a continuous process for the production of CX granules was proposed by Eskenazi et al. [59]. In this process, an aqueous solution of resorcinol and formaldehyde is introduced into a column filled with hot oleic acid. By varying the circulation rate of oleic acid, it is possible to adjust the conditions under which gel formation is completed before the solution passes through the column, which allows the granules to solidify before reaching the bottom of the column. CXs obtained after pyrolysis possess high values of specific surface area and pore volume.

### 3.4. Carbonization Stage

**Figure 7:**
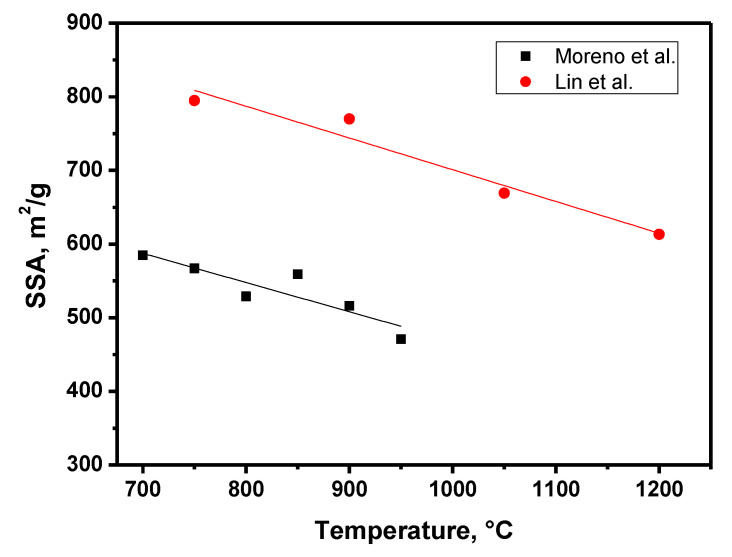
Effects of the pyrolysis temperature on the SSA values reported by Moreno et al. [61] and Lin et al. [62].

The next step in the process of preparing CXs is the pyrolysis stage. It consists of heat treatment in an inert atmosphere (N_2_, Ar) at temperatures of 800–1300 °C. It can be assumed that the mechanism of pyrolysis is similar to that for phenol–formaldehyde resins and involves the formation of water, CO, CO_2_, methane, and other organic compounds [60]. According to differential thermal analysis [61], the mass loss is insignificant at temperatures above 800 °C. Obviously, at this temperature, the decomposition processes of organic precursors are completed, and at higher temperatures, only residual nitrogen is being removed. These processes lead to the formation of porous structures of CXs and, above all, micropores. Table 1 summarizes the effects of the pyrolysis temperature and duration on the properties of CXs. Figure 7 demonstrates that the SSA value decreases almost linearly with the increase in the pyrolysis temperature. It can be concluded that the material properties can be changed controllably by varying these parameters.

### 3.5. Activation of CXs

**Figure 8:**
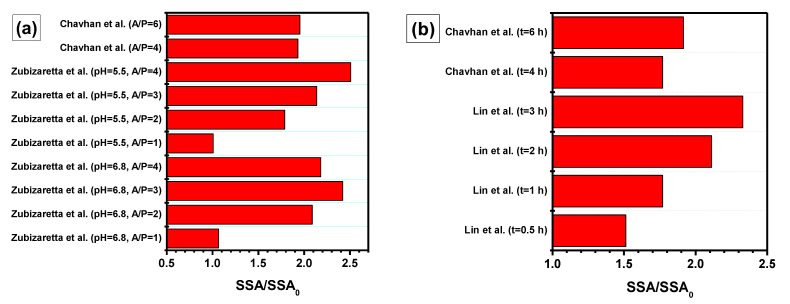
Relative surface area (SSA/SSA_0_) of the CXs: (**a**) activated by KOH; (**b**) activated by CO_2_. The SSA values were calculated from the data reported by Zubizaretta et al. [35], Chavhan et al. [40], and Lin et al. [62].

Extremely high textural characteristics can be achieved via activation of CXs by CO_2_ or alkali (Figure 8). In the case of alkaline activation, the sample of CXs is stirred in a KOH solution at 85 °C until the evaporation of the solution and then subjected to repeated pyrolysis [35]. Chemical activation of carbon materials by hydroxides consists of a series of redox reactions, in which carbon is oxidized with the formation of carbonates, carbon oxides, and hydrogen. In addition, potassium can be released in the metallic state [63]. After pyrolysis, the residual K^+^ ions can be removed by washing. During chemical activation, the volume of micropores increases significantly, but the structure of the carbon material is mainly preserved. Mesopores formed during gel synthesis are not affected by the alkali treatment, and the morphology of activated xerogels does not differ from the morphology of the original samples. It seems that the C-C bonds between the individual particles in the xerogel structure formed in the pyrolysis stage are sufficiently strong and inert to alkali. However, the carbon matrix still becomes more brittle as a result of activation, which leads to the destruction of the monolithic shape of the samples and the formation of powder. Nevertheless, by using alkaline activation, it is possible to achieve more advanced microporosity for each given resorcinol/catalyst ratio and meso-/macropore size. The specific surface area of the activated materials reaches 1800 m^2^/g with a pore volume of ~3 cm^3^/g.

Similar processes occur in the case of the activation of CXs with carbon dioxide. This treatment is carried out at temperatures of 800–1100 °C in a gas flow containing several percent of CO_2_. The CX material reacts with carbon dioxide via the inverse Boudouard reaction (Equation (1)), which leads to the development of porosity. According to [62], an increase in activation time from 30 min to 3 h results in an increase in specific surface area from 698.8 to 1629.4 m^2^/g.

(1) $$C+{CO}_{2}⇄2CO$$

### 3.6. CXs Derived from Other Precursors

As demonstrated above, the sol–gel polycondensation of resorcinol and formaldehyde allows for obtaining CXs with a wide variety of porous structure types. However, it is interesting to consider whether any other precursors could be applied to the preparation of such materials. Indeed, according to the literature data, both resorcinol and formaldehyde can be substituted for other compounds. In certain cases, these compounds may contain heteroatoms, which causes the formation of heteroatom-doped gels and xerogels. Firstly, precursors containing oxygen, carbon, and hydrogen only will be considered.

Phenol–formaldehyde resins were the first commercially available synthetic resins, which are still widely used in industry. The main question here deals with the possibility of finding conditions in which the polycondensation of phenol and formaldehyde will yield porous products. Scherdel and Reichenauer tried to answer this question in their work [64]. The use of an organic solvent (n-propanol) combined with an acidic catalyst (HCl) allowed them to obtain CXs with a specific surface area as high as 572 m^2^/g. The corresponding volumes of mesopores and micropores were 0.69 and 0.17 cm^3^/g, respectively. However, these textural parameters are noticeably lower than those obtained from resorcinol and formaldehyde. Unfortunately, no information regarding the pH values of the precursor solution was reported. At the same time, the effects of such synthetic parameters as the concentration of HCl, mass contents of phenol and formaldehyde, and phenol/formaldehyde ratio on textural properties were carefully investigated. Thus, at mass concentrations of 15 and 25%, lower HCl concentrations favored the formation of products with higher surface areas and pore volumes, but at 35%, the dependence was non-monotonic. In contrast, in alkaline conditions, only non-porous solids were obtained. In general, it is possible to tune the texture like in the case of RF gels but with more limitations.

Under the conditions studied by Jirglová et al. [65], the condensation of phenol and formaldehyde was not possible. However, gels were obtained from mixtures of phenol with phloroglucinol (1,3,5-trihydroxybenzene). Interestingly, the CXs obtained from such mixtures are non-porous, while the carbon aerogels possess well-developed porosity and texture. The structure of the corresponding gels is more fragile compared to RF gels.

Another route to produce porous materials from non-porous commercial phenol–formaldehyde resins is the use of pore-forming agents. For this purpose, Molchanov et al. [66] applied a solution of oxalic acid hexahydrate (30 g) in glycerol (100 g). The obtained materials consisted of globules with a size of 5–10 µm, which exhibited a turbostratic structure formed by chaotically arranged micro-blocks with sizes of 1–2 nm. Various amounts of pore-forming agents were used to obtain CXs with surface area values ranging from 458 to 596 m^2^/g and micropore surface areas ranging from 402 to 541 m^2^/g. This indicates that the CX materials were mostly microporous, and the volume of mesopores was rather low. Still, these materials demonstrated excellent hydrogen adsorption properties. The storage capacity normalized to the surface area was noticeably higher in comparison with commercially available carbon sorbents. Overall, it was demonstrated that phenol can be applied as a cheaper alternative to resorcinol in the synthesis of CXs [64,66]. It should be noted that the activation of phenol–formaldehyde-derived carbons with CO_2_ or KOH allows for obtaining microporous carbon materials with surface areas up to 2200 m^2^/g [67]. However, the tunable meso-/macroporosity remains the unique feature of resorcinol–formaldehyde xerogels.

Another analog of resorcinol, 5-methyl-1,3-dihydroxybenzene (5-methyl resorcinol, MR), can also be used for the production of CXs. In this compound, a methyl group occupies position 5 of the benzene ring. However, positions 2, 4, and 6, which are most active for the electrophilic substitution reaction, are still available for the process. On the other hand, the presence of a methyl group might induce steric hindrance. In fact, it was shown that the gelation of MR and formaldehyde with the formation of porous xerogels is possible under certain conditions [68]. In this case, rather low resorcinol/Na_2_CO_3_ ratios in a range of 30–90 are required. When the Na_2_CO_3_ content is high, the undesirable formation of needle-like crystals can take place. Nevertheless, it was possible to obtain CXs with surface areas up to 417 m^2^/g and electric conductivity of 0.8 S/cm via this route. Therefore, this material seems to be suitable for electrochemical applications as an electrode material. In addition, the properties of MR-formaldehyde xerogels can be tuned by changing the process parameters, like in the case of RF gels.

Another direction of the CX preparation involves using natural phenolic compounds such as tannins and lignins [69,70]. Tannins are produced from various sources, such as larch bark and pine biomass. Due to the presence of phenolic functional groups, these compounds can substitute for resorcinol in the synthesis of CXs. The surface area of xerogels prepared via polycondensation of tannins with furfuryl alcohol reached 585 m^2^/g. This sample demonstrated a benzene sorption capacity of 0.83 g/g, which is comparable to commercial carbon sorbents [70].

Formaldehyde can also be substituted for another aldehyde—furfural [71,72,73]. The mechanism of the polycondensation of furfural and resorcinol involves the formation of α-furfurylhydroxymethyl derivatives of resorcinol and their subsequent condensation, as proposed by Wu et al. [71]. The careful control of the reaction conditions allowed for preparing CXs with appropriate surface areas of 698–753 m^2^/g and large volume values of mesopores (1.09–1.64 cm^3^/g), while the micropore surface areas were comparatively low. In this case, polycondensation was conducted in an ethanol solution, and NaOH was used as a basic catalyst. The framework of the aerogel samples consisted of interconnected nanoparticles of about 20 nm in diameter, which defined numerous mesopores of less than 30 nm. In addition, the polycondensation of furfural and resorcinol can be carried out using acidic catalysis [73]. After the activation of thus-obtained CX materials, their surface area reached 1924 m^2^/g.

## 4. Methods for Doping CXs with Heteroatoms

### 4.1. Nitrogen-Containing Functional Groups

**Figure 9:**
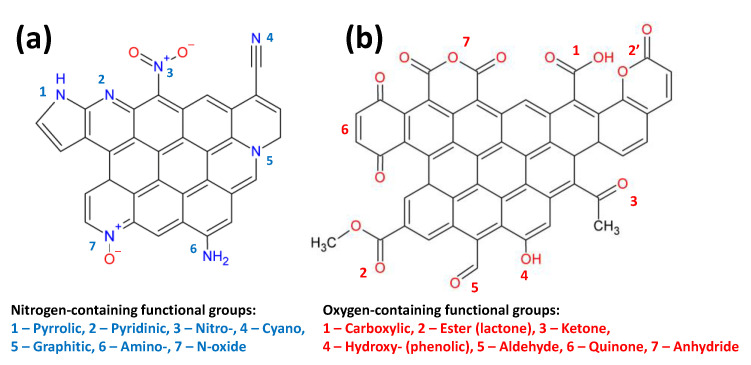
Types of nitrogen-containing (**a**) and oxygen-containing (**b**) functional groups found on the surfaces of CXs.

It is known that modifying carbon materials with heteroatoms significantly increases their efficiency in several processes [74]. Thus, the presence of nitrogen functional groups helps to increase the dispersion of the deposited metal and affects its electronic state. Particularly promising is the use of nitrogen-modified carbon materials in electrochemical processes [75,76]. Figure 9 presents different types of functional groups that can be found on the surfaces of CXs. These groups are pyrrolic, pyridinic, cyano, nitro, and N-oxide, as well as graphitic nitrogen. In the presence of metal, nitrogen can also be in the form of nitrides.

It is worth noting that the composition of functional groups has a significant impact on the characteristics of CXs in these processes. Thus, Morawa Eblagon et al. [77] studied the effect of the composition of functional groups on the catalytic activity of N-modified CXs in the oxygen reduction reaction. The onset potential and the number of electrons transferred were found to increase with the ratio between quaternary and pyrrolic nitrogen species. The current density is linearly correlated with the specific surface area. Although the effectiveness of each type of functional group can be difficult to ascertain, the presence of nitrogen-containing functional groups has been shown to affect the electrical, electrochemical, adsorption, and catalytic properties of carbon materials [78,79,80,81,82].

Thus, the main research task here is to find ways to introduce nitrogen, ensuring the optimal composition of functional groups. Table 2 provides an overview of the methods for introducing nitrogen into the CX structure and the characterization results of the obtained samples by X-ray photoelectron spectroscopy (XPS) and elemental analysis. Two groups of methods used to introduce nitrogen atoms into the structure of CXs can be distinguished:

(1) Methods based on the use of nitrogen-containing precursors in the polycondensation stage;
(2) Methods based on the post-treatment of organic or carbon xerogels.

In the first case, a nitrogen-containing precursor is also introduced into the precursor solution containing the specified concentrations of resorcinol and formaldehyde at a certain pH. Usually, melamine or urea plays the role of such a precursor, but it is also possible to use other compounds having NH_2_- or nitrogen groups in their structures as part of heterocycles, as well as ammonia [75,83,84].

**Table 2:** Summary of the methods reported for the preparation of CXs functionalized with nitrogen.

No.	Sample	Functionalization Procedure	Heteroatom Content, %	Functional Groups by XPS, %	Ref.
1.	N-CXG-1	Addition of urea to precursor solution	EA—2.9 N, 7.7 O; XPS—2.0 N, 10.7 O	39.1—N6, 4.6—N5, 52.8—NG, 3.6—N-O	[84]
2.	Fe-N-CXG-1-TT2		EA—0.9 N, 4 O; XPS—1.1 N, 6.1 O	17—N6, 9.4—N5, 27.9—NG, 21.3—N-O, Me-N—24.5	[84]
3.	NCX-150-2	Addition of melamine to precursor solution	EA—2.97 N	N6, N5, NG—49.2	[85]
4.	Carbon xerogel	Addition of ammonia to the precursor solution	XPS—1.33 N, 14.44 O	37.15—N6, 54.79 -N5+NG, 8.06—N-O	[86]
5.	Fe-doped carbon xerogel		XPS—1.16 N, 15.19 O	40.95—N6, 48.94—N5+NG, 10.11—N-O	[86]
6.	HAM-500	Co-gelation of hydroxyaniline with melamine and formaldehyde	EA—13 N, 9 O; XPS—20 N, 3 O	53—N6, 41—N5, 5—NG	[14]
7.	HAM-700		EA—9 N, 8 O; XPS—11 N, 2 O	44—N6, 34—N5, 22—NG	
8.	HAM-900		EA—6 N, 8 O; XPS—6 N, 1 O	28—N6, 33—N5, 39—NG	
9.	CX_NH_3__950	Post-treatment with ammonia	EA—2.1 N, 4.9 O; XPS—1.7 N, 4.6 O	46.7—N6, 33.8—N5, 19.5— NG	[77]
10.	CX_NH3_650_BM	Post-treatment with ammonia and ball milling	EA—2.6 N, 6.0 O; XPS—1.8 N, 6.2 O	26.4—N6, 62.2—N5, 11.3—NG	
11.	CX_NH3_950_BM		EA—2.2 N, 6.3 O; XPS—1.4 N, 8.0 O	48.9—N6, 32.5—N5, 18.6—NG	
12.	NH_3_ treated CXG	NH_3_ plasma treatment	XPS—8.5 N	N6, N5 and NG	[87]
13.	CX-HMTA6	Addition of hexamethylenetetramine to the precursor solution	XPS—1.26 N, 15.78 O	N6, N5 and NG	[88]
14.	N-CX-8-1000	Sol–gel polycondensation of resorcinol with pyrrole-2-carboxaldehyde	EA—3.8 N, 13.36 O	31—N6, 12—N5, 48—NG, 9—N-O	[89]
15.	CX-750 N	Post-treatment with melamine of the carbonized CX	XPS—2.4 N, 10.4 O	62.2—N6, 37.8—N5	[90]
16.	AX-1000 N	Post-treatment with melamine of activated carbonized CX	XPS—1.8 N, 5.4 O	49.2—N6, 36.8—N5, 14.0—NG	

EA—elemental analysis; XPS—X-ray photoelectron spectroscopy; NG—graphitic nitrogen; N5—pyrrolic nitrogen; N6—pyridinic nitrogen; N-O—nitrogen oxides; Me-N—nitrides.

Gorgulho et al. [15] compared melamine and urea as precursors. Besides the target modification with nitrogen, the introduction of these compounds also affects the porous structure of the resulting materials. In the case of melamine, the total pore volume was lower than in the case of urea, although the achieved volume of micropores was larger. The contribution of mesopores was also lower in the case of melamine. Both precursors are effective in introducing nitrogen into the structure of CXs. In dried organic gels, the nitrogen content reached 9.5 wt%; however, after pyrolysis, the mass fraction decreased to 2–3.5%. According to elemental analysis, urea-derived xerogels contained less nitrogen compared to melamine-derived materials, while the XPS study of these materials showed a greater surface nitrogen concentration. Nitrogen in the CX structure is part of various functional groups: six- and five-membered heterocycles, quaternary amines, and N-oxides. When the pyrolysis stage is performed at a temperature of 600 °C, CN groups are also formed. Interestingly, the presence of nitrogen leads to an increase in the concentration of both basic and acidic groups, as determined by the titration method.

Another nitrogen-containing precursor used for the synthesis of N-doped CXs is 3-hydroxyaniline. This compound is a structural analog of resorcinol (1,3-dihydroxybenzene), so it can be expected to undergo a polycondensation reaction similar to resorcinol. This makes it possible to completely abandon the use of resorcinol as a precursor. The CXs synthesized using melamine, hydroxyaniline, and formaldehyde by Perez-Cadenas et al. [14] had the highest nitrogen content after pyrolysis at 700 °C, which reached 9 and 11 wt% according to elemental analysis and XPS, respectively. Samples of melamine–resorcinol–formaldehyde xerogels synthesized under the same conditions contain 2 times less nitrogen but have a significantly higher specific surface area and pore volume. It is worth adding that, as a rule, with an increase in pyrolysis temperature, the nitrogen concentration decreases, while the portion of nitrogen within the composition of five- and six-membered heterocycles decreases, but the portion of more stable quaternary nitrogen increases.

Among the post-treatment methods, the method of treatment with gaseous ammonia should be mentioned. This method allows not only the introduction of nitrogen functional groups on the surface of CXs but also the modification of their texture. Ammonia affects porosity in a similar way to treatment with CO_2_ and alkalis. It leads to an increase in specific surface area due to an increase in the volume of micropores. Thus, the exposure of xerogels to ammonia at 950 °C increases the specific surface area from 620 to 1647 m^2^/g and the volume of micropores from 0.20 to 0.51 cm^3^/g. This method allows for achieving a nitrogen concentration of up to 2 wt% on the surface with the formation of pyrrole and pyridine fragments, as well as quaternary nitrogen [77].

The structure of CXs being functionalized affects the composition of the introduced functional groups [90]. Thus, during the post-treatment of carbonized CXs with melamine, only pyrrole and pyridine groups are introduced. The same treatment of activated CXs, which possess a large micropore volume, results in the formation of graphitic nitrogen as well. The latter has a positive effect on the activity of the CXs in the oxygen reduction reaction.

In addition to ammonia, other compounds, such as HNO_3_, HCN, N,N-dimethylethanolamine, N,N-dimethylpropanediamine, urea, dicyanodiamine, N,N-dimethylformamide, and polyaniline, can be used in the post-treatment stage to introduce nitrogen-containing functional groups [76,82,91].

Summarizing the data in Table 1, it can be concluded that sol–gel polycondensation involving nitrogen-containing precursors, such as hydroxyaniline or pyrrole-2-carboxaldehyde, makes it possible to obtain materials with the highest nitrogen content. At the same time, the presence of these compounds in the sol–gel synthesis step results in the lower porosity of the products. Among post-treatment methods, the highest nitrogen content was achieved in the case of NH_3_ plasma treatment [87]. Therefore, in order to produce CXs with optimal properties, the type of precursor should be selected with care. It is also worth noting that none of the methods described in Table 1 allow one type of functional group to be selectively obtained. In most cases, pyrrolic, pyridinic, and graphitic nitrogen are observed in different proportions.

### 4.2. Oxygen-Containing Functional Groups

Various oxygen-containing functional groups, namely, carboxyl, phenolic, lactone, and carbonyl groups, quinones, and anhydrides, can be found on the surfaces of CXs (Figure 9). It is worth noting that as-prepared CXs can contain about 3–7% oxygen (at pyrolysis temperatures of 800–900 °C); however, only a small fraction of oxygen is localized on the surface [40]. Therefore, post-treatment techniques are required to introduce oxygen-containing groups to the surface.

It should be emphasized that the analysis of the O1s XPS spectra is rather complicated compared to the N1s region. More information on the composition of functional groups can be obtained by the Boehm titration and temperature-programmed CO/CO_2_ desorption (TPD) methods [92]. The TPD method is based on the fact that when carbon materials are heated in an inert atmosphere, surface groups decompose to form CO and CO_2_. Different functional groups decompose at different temperatures, which allows one to establish the composition of the surface.

Table 3 presents the most common methods for introducing oxygen-containing groups to the CX surface. Oxidizing treatment with acids is usually used to modify the surface of CXs with oxygen-containing functional groups. For instance, Silva et al. [93] used the hydrothermal treatment method with nitric acid. The following features were observed in this research:

(1) The number of functional groups on the surface of the CXs depends on the treatment conditions, including the concentration of HNO_3_, temperature, and loading of carbon material in the hydrothermal treatment stage.
(2) Such textural characteristics of the CXs as SSA and pore volume are not significantly affected when the treatment temperature is quite low (20 °C). At a temperature of 100 °C, the specific surface area grows with increasing HNO_3_ concentration.

Another route to produce oxygen functional groups is treatment with air oxygen [94,95]. Usually, this procedure is conducted at 400 °C. At this temperature, thermally unstable carboxyl groups are not being formed, and the oxidation treatment results in the formation of more stable groups like phenolic hydroxyls. This method seems to be more convenient because it is a one-step procedure that does not require the use of acids. This procedure, however, may cause the textural modification of the materials [17].

Another fast and facile approach to introducing oxygen functional groups is the treatment with oxygen plasma [17]. It mainly introduces carboxylic groups on the external surface of CXs. According to XPS data, just 10 min of treatment with oxygen plasma is enough to achieve 25–35 at % oxygen. However, contrary to treatment with air or acids, no oxygen-containing functional groups are created inside the micropores of CXs.

### 4.3. Functional Groups Containing Sulfur, Boron, and Phosphorus

The flexibility of the sol–gel method, along with the large variety of existing post-treatment techniques, allows for preparing carbon materials containing other heteroatoms in their structure, such as phosphorus, boron, and sulfur (Table 3). In this section, these techniques will be briefly discussed.

The introduction of boron or phosphorus is known to be an effective way to improve the electrochemical properties of carbon materials. In order to introduce boron into the CX structure, the addition of boron-containing compounds, namely, boric acid or phenylboronic acid, has been used [96,97]. As in the case of nitrogen-containing precursors, boron-containing compounds affect the porosity of the resulting materials. Thus, samples prepared with boric acid had a higher mesopore volume and a wider mean mesopore size than samples prepared with phenylboronic acid [96]. Various forms of boron, such as boron carbides and oxides, are found in boron-doped xerogels.

It should be mentioned that the appropriate choice of precursors may allow one to produce CXs co-doped with both nitrogen and boron simultaneously. Wang et al. [97] supplemented this procedure with the addition of graphene oxide to prepare nitrogen and boron co-doped graphene xerogels. The contents of boron and nitrogen reached 2.59 and 3.89 at%, respectively. The presence of heteroatoms helps to enhance the pseudocapacitance and the surface wettability. Therefore, such materials demonstrated excellent performance as electrodes in the electrochemical desalination of brackish water.

Phosphor is mainly introduced into the CX structure via post-treatment methods, for instance, via post-treatment with phosphoric acid and phosphor vapors [98,99,100,101]. The condensation of P from the gas phase results in the filling of micropores and decreases the surface area from 591 to 118 m^2^/g, but the phosphorus content in thus-prepared materials is as high as 11 wt% [101]. Such materials are regarded as effective anodes for Na-ion batteries, demonstrating high reversible capacity and Coulomb efficiency as high as 99.4%. In contrast, treatment with phosphoric acid followed by pyrolysis in an inert atmosphere preserves the texture or even produces P-doped xerogels with a more developed surface area (up to 1166 m^2^/g [102]). The presence of –P-OH groups on the surface of carbon materials creates additional Brønsted acid sites, which are active in acidic catalysis [98]. P-containing groups significantly increase the electrochemical capacitance as well [99].

The treatment of CXs with concentrated sulfuric acid allows one to introduce sulfonic groups on their surface [54]. A similar result can be obtained via the treatment of carbon materials with a diazonium salt (4-benzenediazonium sulfonate) generated in situ from 4-aminobenzenesulfonic acid. CXs modified with sulfonic groups were found to be quite effective in the process of the esterification of glycerol. Materials modified in this way are not inferior in catalytic activity to commercial ion-exchange resins.

**Table 3:** Summary of the methods reported for the preparation of CXs doped with oxygen, boron, phosphorus, and sulfur.

Heteroatom	Functionalization Procedure	Functional Groups	Application	Ref.
Oxygen	O_2_ plasma treatment	Hydroxyl, carbonyl, and carboxyl groups	Photocatalysis (photodegradation of Rhodamine B)	[17,87]
	Liquid-phase oxidation with HNO_3_	Carboxyl, phenolic, lactone, and carbonyl groups; quinones; and anhydrides	Oxidation and hydrolysis processes, fuel cell catalysis	[93,94,95]
	Gas-phase oxidation with O_2_	More thermally stable groups like phenolic hydroxyls		[94,95]
Boron	Addition of boric acid or phenyl boronic acid to the precursor solution	Boron oxides, oxycarbides, boron carbides, and B in the carbon lattice	-	[96]
	Addition of boric acid to the precursor solution	B–C_2_O (60.4), B–N (36.5), B_2_O_3_ (3.1)	Electrochemical desalination of brackish water	[97]
Phosphorus	Post-treatment with H_3_PO_4_	C-P-O and C-O-P species	Dehydration of fructose to 5-hydroxymethylfurfural	[98]
	Post-treatment with H_3_PO_4_	P-O and P-C bonds	Oxygen reduction reaction	[102]
	Gas-phase condensation of red P	-	anode for sodium-ion batteries	[101]
	Post-treatment with H_3_PO_4_	C–O–P-type groups	Capacitor material	[99]
	Polycondensation reaction of tyrosine and pyrrole-2-carbaldehyde using phosphoric acid as the catalyst	Pentavalent tetracoordinated phosphate (PO_4_) and pyrophosphate groups (PO_3_)	Electrode for supercapacitor	[100]
Sulfur	H_2_SO_4_ treatment	-SO_3_H	Glycerol acetylation	[54]
	4-benzenediazonium sulfonate treatment			
	Addition of sulfur-containing aldehydes like 2-thiophenecarboxaldehyde to the precursor solution	C-S-C, other forms of C-S, and some oxidized species like sulfates and sulfonates	Oxygen reduction reaction (Fe-N-S-CX)	[103,104]

To conclude the discussion on the doping of CXs with heteroatoms, the differences in the effects that these dopants have on the properties of CXs should be considered. Both oxygen and sulfur belong to the 16th group of the periodic table. However, sulfur demonstrates a larger variety of oxidation states than oxygen. On the surface of CXs, sulfur is mainly found within the composition of sulfonic groups (-SO_3_H), which demonstrate high acidity [54]. This explains the application of such materials as solid acid catalysts [54]. Sulfur also has a smaller ionic radius compared to oxygen. Therefore, if it is incorporated into the bulk structure of CXs, a higher degree of disorder is expected. Nitrogen and phosphorus can be compared in the same way. In N-doped CXs, nitrogen is mainly in an oxidation state of -3 and forms a large variety of functionalities (Figure 9). Nitrogen has five valent electrons as opposed to the four valence electrons of carbon. Therefore, N-doped CXs demonstrate n-type semiconductor behavior [105]. In contrast, phosphorus is mostly located in higher oxidation states, thus forming polar phosphate groups on the surface of CXs [98]. These groups are known to improve the electrochemical capacitance to a large extent [99]. Similarly to nitrogen, boron is known to improve conductivity and enhance electrochemical properties. However, boron-doped carbons are p-type semiconductors. Thus, the CXs doped with nitrogen and boron present peculiar electronic structures [105].

## 5. Doping of CXs with Metals

### 5.1. Conventional Impregnation-Based Approaches

To obtain supported catalysts based on CXs, all conventional methods used for the preparation of catalysts on other supports can be applied. It is known that the presence of functional groups on the CX surface makes it possible to significantly improve the dispersion of the supporting metal and modify its electronic state [106,107].

The influence of functional groups is most obvious for the deposition of metals by electrostatic adsorption. Lambert et al. [108] reported several CXs prepared at different pH values in the sol–gel synthesis stage. Some of the samples were modified with nitric acid, which allowed the introduction of oxygen-containing groups to the surface. This procedure significantly changes the properties of the CX surface and leads to a change in the pH of the point of zero charge (PZC) of the support. This parameter determines the surface charge of the material and, therefore, the type of ions that will preferentially be adsorbed in the deposition step. Depending on the PZC value, different precursors were used in the deposition stage: anionic (H_2_PtCl_6_) in the case of the as-prepared xerogels and cationic ([Pt(NH_3_)_4_]Cl_2_) for the oxidized ones. Optimal conditions allow one to deposit platinum in the form of fine particles of about 1 nm in size.

It should be mentioned that in the case of activated xerogels having a specific surface area of up to 2000 m^2^/g, even conventional impregnation methods allow for achieving satisfactory metal dispersion. For instance, a catalyst containing 10 wt% Ni at a particle size of 2.7 nm was successfully prepared using activated CX material with a surface area of about 1600 m^2^/g [109].

### 5.2. Approaches Based on One-Step Sol–Gel Synthesis

**Figure 10:**
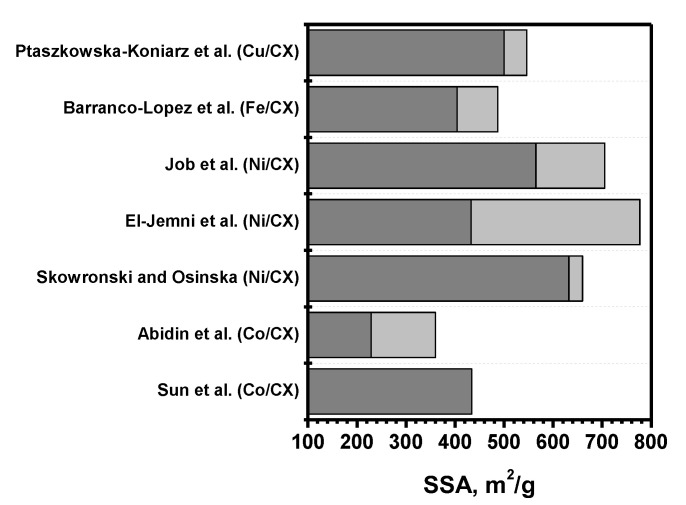
Ranges of SSA values for the metal-doped CXs reported by Sun et al. (Co/CX) [110], Abidin et al. (Co/CX) [111], Skowronski and Osinska (Ni/CX) [112], El-Jemni et al. (Ni/CX) [113], Job et al. (Ni/CX) [114], Barranco-Lopez et al. (Fe/CX) [115], and Ptaszkowska-Koniarz et al. (Cu/CX) [116].

An alternative to impregnation may be the joint synthesis method. In this case, the precursor of the active component is introduced during the polycondensation step or into an already-formed gel. These methods make it possible to simplify the preparation procedure, but the presence of metal in the gel-forming and pyrolysis stages can significantly affect the texture of materials. Table 4 summarizes the synthesis methods and potential applications of materials prepared via such a single-step sol–gel procedure. The general effects of the metal addition are shown in Figure 10.

Sun et al. [110] introduced cobalt nitrate into the non-dried organic gel. The presence of cobalt in the pyrolysis step was found to increase the degree of graphitization of the material and reduce the SSA to 434 m^2^/g (Figure 10). This led to the formation of sufficiently large cobalt particles, whose catalytic activity in phenol oxidation was inferior to that of a sample obtained by the impregnation of an activated CX with a specific surface area of 2600 m^2^/g. It is worth noting that the sample obtained by joint synthesis was not activated with carbon dioxide, which explains such a significant difference in the specific surface area.

Zainul Abidin et al. [111] reported that the introduction of cobalt also affects the value of mass loss in the pyrolysis stage. At the same time, the mass loss decreases with a decrease in pH, which also leads to a decrease in the cobalt concentration at the same loading in the gel-forming stage. Despite this, textural characteristics possess the same features that were considered previously. The difficulty of selecting appropriate parameters for the synthesis of materials with a given metal concentration is one of the disadvantages of the joint synthesis method.

The introduction of nickel in a similar manner to obtain concentrations of 7 and 10 wt% in the final CX also affected the texture [112]. In this case, there was an increase in SSA (Figure 10) due to the growth of the volume of micropores, but at the same time, the total pore volume decreased by 4 times. The X-ray diffraction patterns of these samples show rather narrow nickel reflections corresponding to relatively large particles, although the reflexes of the carbon phase remained expanded when nickel was introduced. El-Jemni et al. [113] also reported that the concentration of introduced Ni has a significant effect on the texture. Thus, at a concentration of 1%, the surface area and pore volume values were 777 m^2^/g and 2.37 cm^3^/g, respectively. At the same time, an increase in concentration to 15% results in almost complete pore blocking, which reduces the surface area to 6 m^2^/g and the pore volume to 0.05 cm^3^/g. The sample containing a medium loading of Ni (6 wt%) with an average size of NiO particles of 18.6 nm demonstrated the most efficient performance in ethanol electro-oxidation, producing a peak oxidation current of 5925 A/gNi.

The texture of metal-doped CXs can be tuned by controlling the pH value as well. However, the pH interval corresponding to the formation of meso-/microporous xerogels depends on the nature of the metal [114]. For instance, Ni-doped, Fe-doped, and Pd-doped mesoporous xerogels were obtained within pH intervals of 6.0–6.75, 4.5–5.8, and 5.55–7, correspondingly. As was mentioned before, the type of cation present in the gel-forming stage often affects the resulting structure. The general trends in the pH effect are similar to those discussed for CXs without metal addition. The metal particles become relatively large (15–200 nm) after drying. They are mainly localized outside of the polymer network due to the reduction of metal cations by formaldehyde. However, by choosing an appropriate complexing agent, it was possible to incorporate Pd and Pt into the xerogel structure [117]. In this case, metals do not sinter noticeably in the pyrolysis stage, and the particle sizes of Pd and Pt were 3–5 nm. However, in the case of Ni and Cu, even when complexing agents were used, the particles were 10 to 40 nm in size. In both cases, the metals were almost completely covered by graphitic layers, and their surfaces were inaccessible to the potential reagents.

As in the case of Ni, the addition of Fe noticeably increases the pore volume but decreases microporosity [115,118,119]. The presence of Fe in the pyrolysis stage results in the formation of graphitized structures near the metallic particles. Therefore, the addition of metal ions can be considered as another way to control the properties of CXs. This contributes to the higher electrocatalytic activity of the Fe-doped CXs. The simultaneous addition of Fe acetate and urea allows the preparation of Fe-N/CX catalysts with improved activity toward the oxygen reduction reaction [84].

Chen et al. used an Fe-containing deep eutectic solvent (a 1:1:4 mixture of choline chloride, iron chloride, and ethylene glycol) as a medium for the synthesis of RF organic gels [118,119]. The presence of Fe during pyrolysis has led to a high graphitization degree. The resulting CX material was purified from iron via washing with hydrochloric acid. The combination of high microporosity and a high graphitization degree makes these carbons excellent candidates for anode materials in Li-ion batteries.

By adding two different metal salts to the precursor solution, it is possible to prepare CXs containing these two metals. Skowroński and Osińska managed to prepare Ni-Pd/CX bimetallic electrocatalysts via this route [120]. However, no alloying of metals was observed, and Ni and Pd formed separate phases. In general, it can be assumed that bimetallic catalysts of the alloyed type can also be prepared by the sol–gel method, but additional investigations are still required.

As follows from the data described above, the presence of metals capable of forming carbides (nickel, cobalt, and iron) in the pyrolysis stage leads to the shrinkage of the porous structure and an increase in the graphitization degree. On the other hand, it can contribute to an increase in microporosity. In this regard, it is interesting to consider the possible effects of metals with a lower affinity for carbon. Ptaszkowska-Koniarz et al. [116] applied the joint synthesis method to obtain a Cu/CX catalyst with a rather high pore volume of 1.16 cm^3^/g and a specific surface area of 546 m^2^/g. The reflections of the carbon phase remained expanded when copper was introduced and retained their positions. Thus, the presence of copper does not increase the graphitization degree. It is worth noting that nitrogen-containing compounds can be introduced together with the precursor of the active component, which makes it possible to obtain modified Cu/N-CX materials in one step.

**Table 4:** Selected applications in catalysis and adsorption of metal-doped CXs prepared via one-step sol–gel synthesis approach.

No.	Material	Preparation Method	Application	Ref.
1	Co-CX	Doping Co^2+^ ions into wet organic gel	Heterogeneous oxidation of phenol	[110]
2	Co-CX	Solubilization of Co(NO_3_)_2_ in the precursor solution	Oxygen reduction reaction	[111]
3	Ni-CX	Solubilization of nickel acetate in the precursor solution	Electrode materials for supercapacitor	[112]
4	Ni-CX	Solubilization of nickel acetate in the precursor solution	Ethanol electro-oxidation	[113]
5	Fe-CX	Solubilization of iron acetate in the precursor solution	Electro-reduction of O_2_ to H_2_O_2_ combined with H_2_O_2_ catalytic decomposition	[115]
6	Cu-N-CX	Solubilization of copper acetate in the precursor solution	Adsorption of caffeine	[116]
7	Fe-N-CX and Fe-N-S-CX	Solubilization of iron chloride in the precursor solution	Oxidation and adsorption of chlorophenols	[121]
8	Co-N-CX	Solubilization of cobalt acetate in the precursor solution	Oxygen reduction reaction	[122]

## 6. Application of Carbon Xerogels

**Figure 11:**
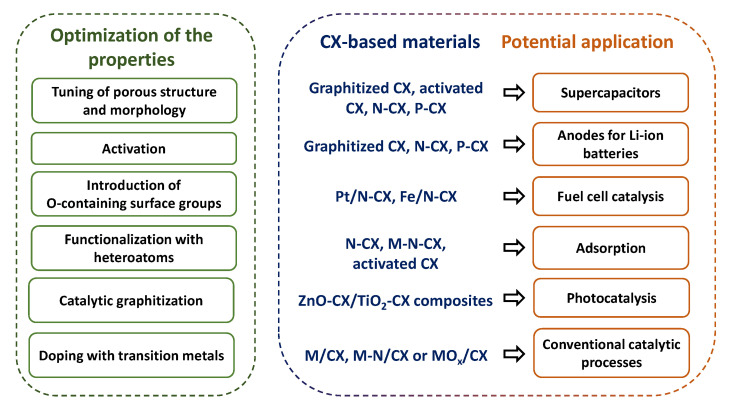
The variety of CX-based materials, their applications, and approaches to optimize their properties.

Finally, the potential areas of application of CXs will be discussed. As shown in Figure 11, these areas are quite diverse and include Li-ion batteries, supercapacitors, fuel cell catalysts, photocatalysis, adsorbents, etc. In each case, the performance of CXs can be tuned to obtain maximum efficiency.

### 6.1. Anodes for Li-Ion Batteries

Li-ion batteries are still regarded as one of the most efficient energy storage devices. Hard carbon materials, like graphite, have been used as anode materials since the 1990s. However, graphite-based electrodes often suffer from a low energy density and poor rate capability [45]. The anode reaction taking place in Li-ion batteries involves the intercalation of graphite with Li ions according to the following stoichiometry:

(2) $${Li}^{+}+6C⟶{LiC}_{6}$$

Because of their high microporosity, surface area, and disordered structure, CXs can incorporate large numbers of Li ions between graphitic layers that directly influence their performance. In fact, the reversible capacity was found to correlate with the microporosity of CXs [123].

The performance of CXs can be significantly improved via an increase in the graphitization degree. The graphitization stage can be realized efficiently in the presence of metals (Ni, Co, and Fe) [124,125,126], as described in the previous section. A capacity of 578 mAh/g was achieved for graphitized CXs after 210 cycles at 0.2 C with a coulombic efficiency greater than 99% [125].

Composite anode materials, such as Si/C, have been viewed as potential candidates for the new generation of Li-ion batteries [127]. A CX/silicon composite with 30 wt% silicon presents a specific discharge capacity as high as 917 mAh/g after 200 cycles and excellent long-term stability at a high current density [128].

### 6.2. Supercapacitor Materials

Supercapacitors are prospective materials for energy storage that utilize an electrical double layer. CXs are regarded as potential double-layer supercapacitors due to their high surface area and hierarchical micro-/meso- or micro-/macroporosity [129]. The performance of CXs as supercapacitors can be greatly improved via activation procedures that increase the surface area, as described above. The flexibility of the sol–gel process allows the application of various modifications, which can help to improve capacitance. Such modifications include the addition of metal ions catalyzing the graphitization process during the carbonization stage and the addition of carbon nanotubes and graphene oxide along with nitrogen-containing precursors [119,130,131]. Thus, Fe-assisted graphitization allows one to obtain CXs that demonstrate a specific capacitance of 209 F/g at a current density of 0.5 A/g, an excellent rate performance (131 F/g at 10 A/g), and appropriate cycling stability [119]. Composite supercapacitor materials, such as CX/polyaniline and CX/NiCo_2_O_4_, can be prepared via post-modification of as-prepared CXs [132,133]. Among hybrid composite materials, the best supercapacitor performance was shown by silver-doped CXs functionalized with 1 wt% nickel cobaltite. NiCo1/Ag-CX exhibited a high specific capacitance (368 F/g at 0.1 A/g), a low equivalent series resistance (1.9 Ω), a high rate capability (99% capacitance retention after 2000 cycles at 1 A/g), and high energy and power densities (50 Wh/Kg, 200 W/Kg at 0.1 A/g) [133].

### 6.3. Fuel Cell Catalysts

In electric fuel cells, CXs can be used as both cathode and anode materials. Recently, of particular interest was the possibility of substituting Pt-based catalysts used for the oxygen reduction reaction (ORR, cathode reaction) and methanol oxidation (anode reaction) for metal-free catalysts or catalysts containing non-noble-metal catalysts. Thus, iron and nitrogen co-doped CXs prepared via a one-step sol–gel polymerization approach demonstrated performance in the ORR close to that of commercial Pt/C catalysts [90,134]. For instance, iron- and nitrogen-doped CXs exhibited outstanding stability, with a half-wave potential of 0.692 V in an alkaline solution (0.1 M KOH), as well as a limiting current density of −31.5 mA/cm^2^ at 0.17 V vs. a reversible hydrogen electrode [134]. The active sites for such catalysts are believed to be Fe-N_x_-C species. Mixed oxides and sulfide-containing CX-based catalysts demonstrate rather efficient performance in the ORR and methanol oxidation [135,136,137]. One such material, MoS_2_/CX, is reported to demonstrate a high current density of 550 mA/cm^2^, along with excellent long-term stability.

### 6.4. Adsorbents

Due to their high surface area, micropore volume, and tunable texture, CXs find wide applications for various adsorption processes. They demonstrate excellent performance for crude oil removal from oil-in-saltwater emulsions [138] and possess a large adsorption capacity for the removal of naphthenic acids from oil sands process water [139,140]. CXs prepared at pH = 5.5 showed an equilibrium adsorption capacity of 15 mg/g in relation to acid-extractable fractions and 7.8 mg/g in relation to naphthenic acids. When adsorbate molecules are large, mass transfer limitations play a crucial role in kinetics, and therefore, the porous structure must be tuned accordingly.

CXs are also regarded as prospective sorbents for effluent treatment because they demonstrate high adsorption capacities toward dyes and metal ions [6,141]. It is important to note that such sorbents are regenerative and reusable. Interestingly, non-carbonized organic xerogels perform more efficiently in the removal of metal ions compared to CXs due to a higher oxygen content on the surface [142]. Clay-templated xerogels demonstrated the highest sorption capacity toward diclofenac because of their optimal porous structures [142]. CX materials have also been applied for the adsorption of various compounds, such as metronidazole [143], caffeine [116], thymol blue [144], and Rhodamine B [145]. Particularly efficient performance was achieved in the case of CXs modified with copper- and nitrogen-containing functional groups. In this case, the adsorption capacity toward caffeine was reported to be as high as 91–118 mg/g.

CXs are applicable for the removal of contaminants from various gas mixtures. Thus, activated CXs were shown to efficiently remove benzene from contaminated air streams [146]. In this case, the samples were activated using NH_4_Cl instead of KOH. The obtained highly microporous sorbent demonstrated reproducible performance in seven adsorption/desorption cycles, and the adsorption capacity toward benzene was more than 2000 mg/g. Because the adsorption capacity and kinetics for various gases are different, CXs can be used for selective adsorption. For instance, the adsorption capacity and adsorption heat values are higher in the case of CO_2_ (6.57 mol/kg and ΔH_ads_ = −28.4 kJ/mol) compared to those of CH_4_ (3.83 mol/kg and ΔH_ads_ = −19.6 kJ/mol) [147]. This allows the application of CXs for the efficient separation of these gases. Therefore, the removal of CO_2_ using CXs is effective for biogas upgrading. Due to their developed porous structures, such materials show no mass transfer limitations, which is quite important for their application in separation processes. In gas chromatography, CXs demonstrate adsorption properties similar to those of other carbon materials [148]. They can be applied for the analysis of light hydrocarbons and the gaseous products of many catalytic reactions.

### 6.5. Photocatalytically Active Composite Materials

Nowadays, heterogeneous photocatalysis is considered one of the most efficient strategies for wastewater treatment. Semiconductor materials are used in such photocatalytic processes. The irradiation of semiconductor materials with UV or visible light leads to the formation of electron–hole pairs, which generate active species (free radicals). These radicals can react with organic contaminants, resulting in their complete oxidation. Thus, photocatalytic performance can be improved by coupling a superconductor material (ZnO, TiO_2_, g-C_3_N_4_, etc.) with a carbonaceous support/adsorbent possessing a high adsorption capacity toward the target organic contaminants. In recent years, CXs have been utilized as such adsorbents due to their tunable porosity and high surface area [87,149,150,151,152,153,154,155]. These materials were used for the removal of Rhodamine B, methylene blue, 4-chlorophenol, bisphenol A, and acetaminophen from water. The ternary composite ZnO/g-C_3_N_4_/CX with an optimal composition showed 92% and 72% degradation of 4-chlorophenol under solar and visible radiation, correspondingly. Such composite materials can be synthesized via a one-step co-gelation approach [150,151,153]. The photocatalytic performance of the materials can be improved by tuning the composition of the catalysts as well as by introducing functional groups on the surface of CXs [149].

### 6.6. Other Catalytic Processes

Carbon materials are widely used as catalyst supports, and CXs are not an exception. They have been investigated in several conventional heterogeneous catalytic processes, including the dehydrogenation of decalin for hydrogen production [156], selective dehydrogenation [157,158], NO_x_ reduction [50,159], and ammonia decomposition [160]. For instance, a Cu/CX catalyst showed 100% conversion of NO_x_ to N_2_ with 100% selectivity at 230 °C and, thus, exceeded the performance of Cu/BEA and other copper-modified carbons [159]. L-Asparaginase was immobilized on RF-derived CXs to catalyze L-asparagine hydrolysis [161]. Despite there being few publications on these topics, the widening of the application areas of CXs in heterogeneous catalysis is expected in the near future.

## 7. Conclusions

Several conclusions can be drawn from the given review. The use of the sol–gel process of polycondensation of resorcinol and formaldehyde for the production of organic xerogels followed by pyrolysis is a promising method for the production of carbon materials. However, due to the nature of the chemical reactions taking place in the polycondensation stage, the process is highly influenced by such parameters as pH, the concentrations of reagents, their ratio, and temperature. A lot of research works devoted to the synthesis of RF gels have shown that variation in these parameters, as well as the drying and pyrolysis conditions, allows one to controllably change the properties of the resulting materials within a wide range. For instance, the pore size can be varied in a range from 2–3 nm to 100–200 nm. The pore volume and SSA values can be as high as 2 cm^3^/g and 1000 m^2^/g, respectively. The use of organic solvents and surfactants makes it possible to obtain carbon materials with various types of morphologies, including carbon microspheres. The synthesis technique can be easily modified to produce nitrogen-, boron-, and phosphorus-containing CXs. Various post-treatment methods allow the introduction of functional groups without leading to the deterioration of textural and structural characteristics. The highest amount of nitrogen can be incorporated into the structure of CXs via co-gelation approaches involving the use of nitrogen-containing precursors. Alkaline and CO_2_ activation of CXs makes it possible to achieve specific surface areas as high as 2000 m^2^/g. The active metal components can be deposited on the surface of modified CXs and stabilized in a highly dispersed state. Nevertheless, methods based on the introduction of the precursor of the active component in the gelation stage remain promising due to their technological simplicity. In some cases, such methods make it possible to obtain materials with properties that exceed those of analogs obtained by impregnation techniques. The presence of metal within the xerogel composition at the pyrolysis stage allows one to produce materials with a higher degree of graphitization.

All of the methods mentioned above can be used to fine-tune the properties of materials in order to achieve maximum efficiency in the target processes. The sol–gel method can be modified to obtain a wide variety of composite materials with specialized properties due to the introduction of metals, non-metal heteroatoms, oxides, and other inorganic components. This explains the wide application areas of CX-based materials: from Li-ion batteries, supercapacitors, and fuel cells to adsorbents and (photo)catalysts. Yet, the scientific fundamentals of the preparation of such materials are to be developed. During the sol–gel process, inorganic precursors can interact with organic monomers, thus leading to the incorporation of these precursors into the gel structure. Therefore, an optimal combination of synthetic parameters must be found in order to obtain materials with the target structure and the uniform distribution of components. The use of modern physicochemical methods, such as high-resolution transmission electron microscopy, X-ray photoelectron spectroscopy, and X-ray absorption fine-structure and X-ray absorption near-edge structure spectroscopies, combined with computational techniques, is required to reveal all of these interactions between the constituents of the composites. Therefore, it is expected that in the near future, the interest of researchers in CX materials will increase intensively.

## Figures and Tables

**Table 1:** Influence of the pyrolysis conditions on the properties of CXs [8,40,61].

Factor	Effect
Increase in the pyrolysis temperature	Decrease in the oxygen concentration
	Decrease in the specific surface area (if particle size exceeds 212 microns) and pore volume
	Increase in macroporosity
	Increase in microporosity at low R/C values (high pH)
	Rise in the electrochemical capacity at temperatures up to 850 °C (decrease at higher temperatures)
	Decrease in the interspace distance (d002)
	Rise in the electric conduction within a temperature range of 500–700 °C (no effect within a range of 700–900 °C)
Increase in the pyrolysis duration	Increase in the specific surface area, pore diameter, and volume
	Rise in the electrochemical capacity at times up to 3 h (decrease at longer times)
Increase in the heating rate	Insignificant increase in the specific surface area

## Data Availability

Data is contained within the article.
